# Mulberry branch bark powder significantly improves hyperglycemia and regulates insulin secretion in type II diabetic mice

**DOI:** 10.1080/16546628.2017.1368847

**Published:** 2017-09-06

**Authors:** Xiao-Lu Yin, Hua-Yu Liu, Yu-Qing Zhang

**Affiliations:** ^a^ Silk Biotechnology Laboratory, School of Biology and Basic Medical Sciences, Soochow University, Suzhou, P R China; ^b^ National Engineering Laboratory for Modern Silk, Soochow University, Suzhou, P R China

**Keywords:** Mulberry branch bark *powder*, oral administration, hyperglycemia, insulin, dose-dependent effect

## Abstract

This experiment, based on the previous study on *R. mori*, introduces whole mulberry branch powder into the diet to treat diabetic mice. Mulberry branch bark powder (MBBP) was administered orally to streptozotocin (STZ)-induced type II diabetic (T2D) mice to investigate hypoglycemic effects. After a 4-week period of diet consumption containing 5%, 10% and 20% MBBP, the fasting blood glucose, body weight and the related western blotting were measured, pathologic and immunohistochemical were observed. The 20% MBBP group showed a significant reduction in hyperglycemia and hyperinsulinemia; fasting blood glucose and insulin decreased from 25.0 to 14.8 mmol/L and 26.5 to 16.0 mU/L, respectively. Pathologic and immunohistochemical observation showed that MBBP administration lead to the repair of pancreas cells and restoration of insulin secretion. Dietary MBBP was associated with the decrease in the contents of 3, 4-methylenedioxeamphetamine, 8-OHdG, aspartate aminotransferase, and alanine aminotransferase, and the increase in antioxidative ability and glucose tolerance. Western blotting (WB) analysis suggested that MBBP decreased the TNF-α levels, thus relieving inflammation and improving liver function. It also led to the downregulation of apoptosis factor expression. WB also confirmed that MBBP enhanced the gene expression of the key enzymes: insulin receptor, insulin receptor substrate, *p-AKT, GSK3β,* glycogen synthase, G6Pase and phosphoenolpyruvate carboxykinase, which are related to glucose metabolism in the liver, and increase the expression of the genes *PDX-1, GLUT2, MafA,* and glucokinase, related to insulin secretion. Thus, oral administration of MBBP regulated insulin secretion and effectively maintained normal levels of glucose metabolism in mice, which may be done by improving the antioxidant capacity and activating insulin signaling with T2D..

## Introduction

Diabetes, a common endocrine disorder disease, has become a serious threat to human health. Type II diabetes (T2D) accounts for more than 90% of the total number of people with diabetes, and there is an increasing trend toward the development of the disease at progressively younger ages [1]. Patients with diabetes often use drugs to control blood sugar levels, and regulate lipid metabolism in clinical practice, but this has its limitations. It often leads to more serious side effects and adverse reactions such as low blood sugar [2]. With the rise of ‘green medicine,’ many research studies are being devoted to the search for safer and more effective drugs from natural biomaterials to control and treat diabetes.

The cultivation of *Morus L*., an edible medicinal plant, has a history of nearly 4000 years in China, and there is also rich cultivation of this plant in many areas of the world. Other than the use of the mulberry leaf as a food for silkworms, its various parts, such as the root *Cortex mori* [3], stem or branch *Ramulus mori* [4], leaf *Folium mori* [5,6], and fruit *Fructus mori* [7], have many medicinal values, which have brought it to the attention of the modern drug industry. All parts of the plant can lower blood sugar and fat to a greater or lesser extent. In addition, the yield of mulberry leaf and branch is high, but most is wasted as agricultural trash or firewood [8]. This has led to increasing attention being given to the waste of *R. mori*. Various medicinal components, monomer compounds, and functions have been gradually found. Many investigations have found that the stem or branch, especially the bark, contains flavonoids, polysaccharides, alkaloid, resveratrol, volatile oil, phenolic acids and other bioactive compounds.

In earlier studies from our laboratory, the bioactivity of the *R. mori* bark in a mixture of ethanol and water was investigated. First, the capability of the extract to reduce serum insulin and insulin resistance was shown; however, the results also showed that the purer the mixture, the lower the bioactivity to inhibit glucosidase [9,10]. More recently, we have studied the potential prevention effect of MBBP against T2D in mice, and the results have clearly illustrated that MBBP may be a potential candidate as a health food in the prevention of diabetes [10]. Therefore, to further and deepen the research on MBBP, the experiment in this study mainly focuses on the oral administration of MBBP for treating diabetes and on investigating its possible mechanisms of action related to glucose metabolism and insulin secretion.

## Materials and methods

### Materials and sample preparation

The branches of the mulberry cultivar HuSang 32 from *Morus multicaulis* L in the Mulberry Garden of Soochow University, Suzhou, China, were collected in November 2013. Streptozotocin (STZ, S0130) was purchased from Sigma-Aldrich Fine Chemicals, USA. All other solvents and chemicals used were of analytical grade. The mulberry branch was cleaned and dried, and the phloem was extracted. It was then crushed coarsely, before further processing by high-speed smashing, and finally crushed to a superfine powder by a high-speed powder machine, producing a particle size of 100 ˜ 150 microns.

**Figure 1.**
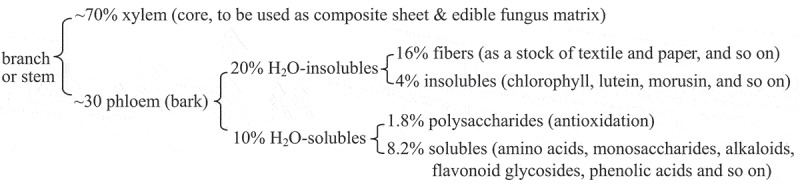
Main components (Morus multicaulis L) of mulberry branches. Mulberry cultivar: HuSang 32.

Recently, the basic composition of the mulberry branches and some bioactive components have been explored and reported by our research group [11–15]. The branch of mulberry cultivar HuSang 32 from *Morus multicaulis* L (Figure 1), was structurally divided into xylem and phloem (or bark), the former accounted for about 70%, for use as composite sheet and edible fungus matrix. The latter accounted for about 30%, it consisted of 20% water-insolubles and 10% water-solubles. The 20% water-insolubles contained 16% fibers and 4% insolubles including chlorophyll, lutein, morusin, and so on; the 20% water-solubles included antioxidative polysaccharides and amino acids, monosaccharides, alkaloids, flavonoid glycosides, phenolic acids, raffinose, arabinose, xylose, and so on. Therefore, we found that except for the 16% of fibers, there were many bioactive components; about 14% of the whole branch mass including water-soluble and water-insoluble matter in the bark of the branch *R. mori*. In other words, the MBBP used in the experiment contained almost one half mulberry bark fibers, and bioactive components in the other half.

### Animal rearing and FBG measuring

A clean grade of 70 male ICR mice (SPF) with body weights (BW) ranging from 12 to 14 g were used in the experiment. All animal experimental protocols used in this study were approved by the Animal Ethics Committee at Soochow University [number of animal license: 201504A136]. The mice were kept under standard laboratory conditions (18–25°C, humidity 50%–80% with a cycle of 12h light and 12h dark) and allowed to feed and drink freely. After rearing with a normal diet for three days, the normal group (10 mice) was reared with a normal diet and the others (60 mice) were reared with a high-fat diet containing 59% basic fodder (05032), 20% sugar, 18% lard oil, and 3% egg yolk, and allowed to feed and drink freely. After one week, STZ was injected into the 60 mice through the caudal vein. STZ (100 mg/kg.BW) was dissolved in cold 0.1M citrate buffer (pH4.4) that was always freshly prepared for immediate usage. After injection for one week, their blood glucose levels were measured when the blood sugar of the mice had become stable, after fasting for 10 h. Fasting blood glucose (FBG) levels ≥11.1 mmol/L were considered T2D diabetic mouse. These diabetic mice were randomly divided into four groups, each group containing 10 mice. One model group was reared with a normal diet, and three treated groups were reared with normal diets containing 5% MBBP, 10% MBBP, and 20% MBBP, respectively. Over four weeks, the growth condition of the mice in each group was observed every day, the average weight of each group was measured every week, and each group of mice was fasted overnight once every week. The FBG levels were measured with a portable OneTouch glucometer (from Johnson & Johnson Medical (Shanghai) Ltd, China), using a drop of blood from the tail vein. After the four-week treatment, the eyeballs of the mice were extracted, and blood was drawn and they were then sacrificed. Their liver, pancreas, kidney, spleen, and testis were excised and weighed. For all the animals in this experiment, operating methods comply with the international animal welfare committee requirements of relevant laws and regulations and provisions.

### Blood lipid determination

The total cholesterol (CHOL), triglyceride (TG), high density lipoprotein cholesterol (HDLC) levels, and low density lipoprotein cholesterol (LDLC) levels were determined for assessing blood lipid level using a BS-800 Chemistry Analyzer (Mindray Medical International Ltd, ShenZhen, China).

### AST, ALT, and 8-OHDG

AST and ALT levels in serum were tested using an AST kit (microplate test, C0102) and ALT kit (microplate test, C0092). The serum of the mice was prepared first and added to the sample in accordance with instructions of the kits; and the absorbance value was measured with SpectraMax M5 (Molecular Devices, Shanghai, China). In addition, the 8-OHDG level was measured using kit protocols (Nanjing Jiancheng Bioengineering Institute, China).

### Antioxidant activity

Weighing 80 mg liver tissue samples in 900 μL ice normal saline and was homogenized, then centrifuged in 4000 r/min, 5 min and reserved liquid supernatant. Antioxidant enzymes, including glutathione peroxidase (GSH-Px) and total superoxide dismutase (T-SOD) activities in the liver tissue, were measured with commercial kits (Nanjing Jiancheng Bioengineering Institute, China). The lipid peroxidation level of the liver tissue was determined according to the content of MDA generated. MDA level assay in the liver tissue was also performed using kit protocols (Nanjing Jiancheng Bioengineering Institute, China).

### Oral glucose tolerance test and insulin tolerance test

For the oral glucose tolerance test (OGTT) the ICR mice were fasted overnight for 12 h. A small tail cut was made to measure the blood glucose using a OneTouch glucometer (from Johnson & Johnson Medical (Shanghai) Ltd, China) as the starting blood glucose value (0 min). Following oral feeding of glucose (2g/kg mice body weight), blood glucose was repeatedly measured at 30 min, 60 min, 90 min, and 120 min. For the insulin tolerance test (ITT) the mice were fasted for 4 h and injected with insulin (0.75 IU/kg mice body weight). Blood glucose levels were determined by tail vein sampling at the indicated intervals (0, 30, 60, 75, 90 min) using a OneTouch glucometer.

### Preparation of pathological tissue

The pancreas was quickly removed from the mice, washed with normal saline, and then dried, weighed, cut into small pieces, and finally fixed with 10% formalin. Tissue dehydration was performed with increasing concentrations of acetone. The sample was then cleaned with xylene and embedded, and 3-μm thick slices were then cut on an HM340E microtome, stained with H&E and insulin immunohistochemical staining, and the histocyte structure and expression of insulin protein were imaged under an optical microscope.

### Western blot analysis

Total proteins from pancreatic tissue were extracted and separated with a 10% SDS-PAGE. Proteins were then transferred to polyvinylidene difluoride membranes (Millipore, Shanghai, China). The membranes were first blocked with 5% defatting milk for 2 h at room temperature and then incubated with monoclonal antibodies against PDK-1 (1:1000), PI3K (1:1000), AKT (1:1000), phosphorylated AKT (P-AKT) (1:1000), GLUT4 (1:1000), G6Pase (1:1000), PEPCK (1:1000), GK (1:1000), PPARγ (1:1000), NFκB (1:1000), TNF-α (1:1000), MafA (1:1000), GLUT2 (1:1000), Bax (1:1000), Bcl-2 (1:1000), and Caspase-3 (1:1000) and GAPHD (1:3000) overnight at 4℃. After being washed, the membranes were incubated with an appropriate secondary antibody (1:5000) for 1.5 h at room temperature. Bands were visualized using a UVP detection system. Band intensity was quantified using LAB WORKS4.6.

### Statistical analysis

We recorded and analyzed experimental data using Origin 7.5 software. The results are reported as mean ±SD and ANOVA were used to evaluate the difference between multiple groups, with a *P* value less than 0.05 considered as significant and a *P* value less than 0.01 as very significant.

## Results

### Change in body weight

**Figure 2.**
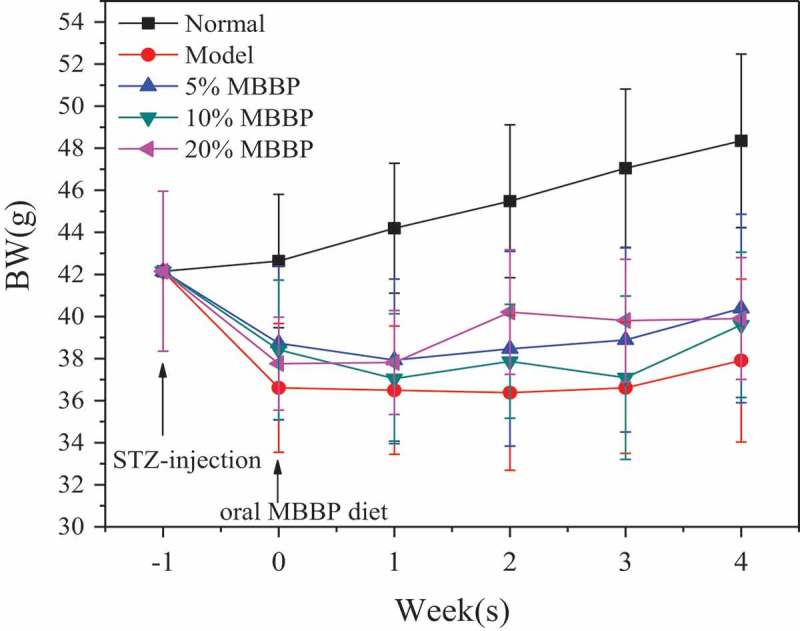
The effect of MBBP on the body weight of mice.

During the test, all the mice were in the growth phase, and the changes in BWs suggested that MBBP had some influence on diabetic mice weight gain. As shown in Figure 2, the BWs of normal mice increased linearly and were stable. During the first week, we found that the BWs of STZ-induced diabetic mice decreased significantly. There were no significant changes in BW between the 5%, 10%, and 20% MBBP treatment groups. In the second week, the BW of 20% MBBP group mice was shown to be higher than that of the 5% and 10% MBBP groups; all model groups had lower BWs than the normal group. At the third and fourth week, the BWs of the three MBBP groups appeared to have no significant difference, but the BW of the model group was lower than that of these MBBP groups. When reviewing the BW changes across the four-week study period, it had been found that the BW of the normal group increased steadily, but the BWs of all MBBP-treated groups decreased. Within the MBBP groups, the BWs of all three dose groups showed no significant reduction, but did not increase either; the weight of the 20% MBBP group, however, was slightly higher than that of the other dose groups. The results showed that STZ-induced diabetic mice lost weight, while the MBBP treatment induced weight restoration to a small degree in the diabetic mice.

### Fasting blood glucose level

**Figure 3.**
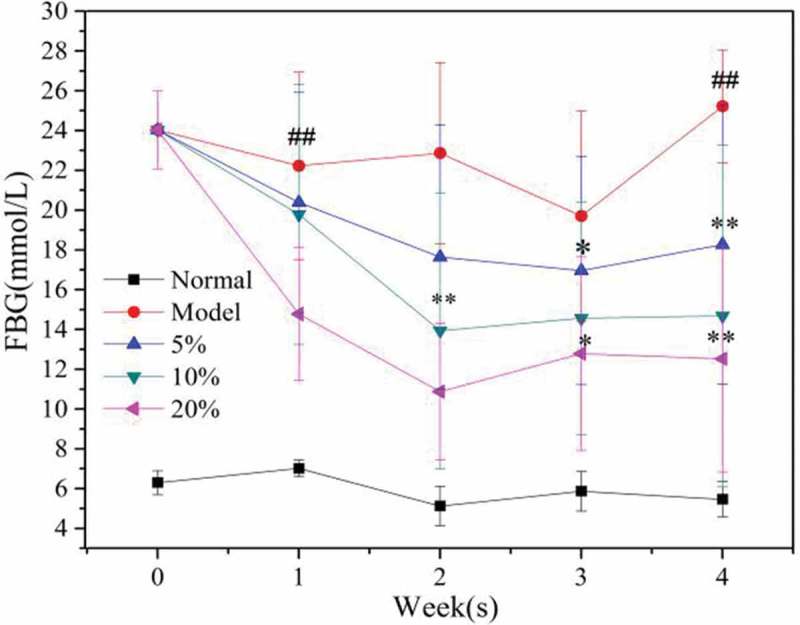
The effect of MBBP on the blood glucose level of STZ-diabetic mice.

In order to observe the effect of oral MBBP on diabetes, blood glucose level was evaluated as standard. The FBG level of the normal group remained stable at 5.1–7.0 mmol/L and its level fluctuated only slightly during the four-week study period, as shown in Figure 3. The FBG of the model group fed with a high-fat diet was significantly higher than that of the normal group, and the highest FBG peaked at 25 mmol/L. After the diabetic mice were fed with MBBP for one week, the blood glucose levels of all three dose groups were significantly decreased; the FBG of the 20% MBBP group decreased substantially and was significantly lower than the model group and the other dose groups. In the third and fourth week of the study period, the blood glucose of every group was stable and showed no significant fluctuation compared with the model group. With the addition of MBBP, the blood glucose of the mice gradually decreased; the FBG of the 20% MBBP group approached 12 mmol/L. The results showed that MBBP had a positive effect on reducing blood glucose in diabetic mice.

### Serum insulin level

**Figure 4.**
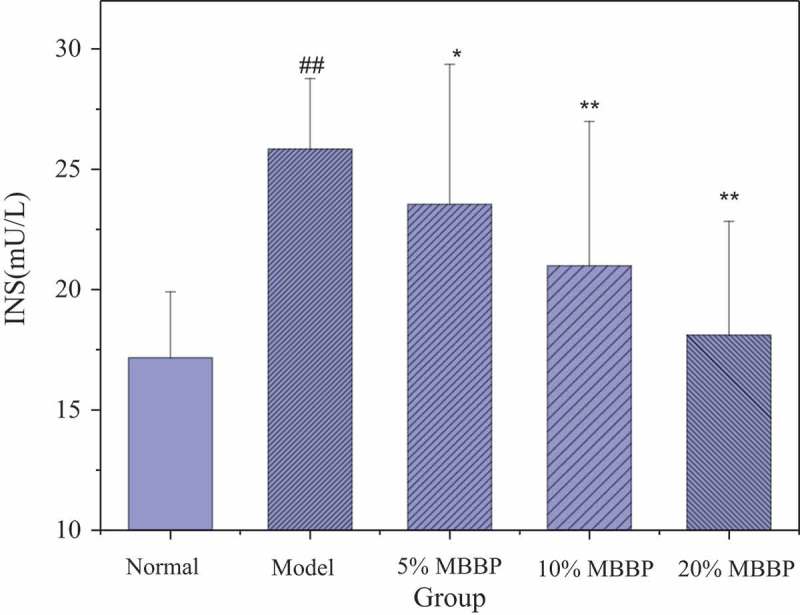
The effect of MBBP on diabetic mice insulin level. ## *p < *0.01 versus the normal group; * *p < *0.05 and ** *p < *0.01 versus the model group.

The abnormal secretion of insulin from islet β-cells is a typical pathological characteristic. As shown in Figure 4, the serum insulin of the model group was significantly higher than that of the normal group (*P* < 0.01). With the three different doses of MBBP treatment for four weeks, the serum insulin level of these diabetic mice reduced significantly in comparison with the model diabetic group. It showed significant changes in the 5% MBBP group (*P* < 0.05), and decreased dramatically in the 10% and 20% MBBP groups compared with the model group, to a very significant degree (*P* < 0.01). The serum insulin levels in the 20% MBBP group were about 17.5 mU/L and returned to levels similar to the normal group. These results showed that the administration of MBBP reduced the serum insulin level effectively and improved insulin resistance in STZ-diabetic mice with a dose-dependent effect.

### 8-OHDG level

**Figure 5.**
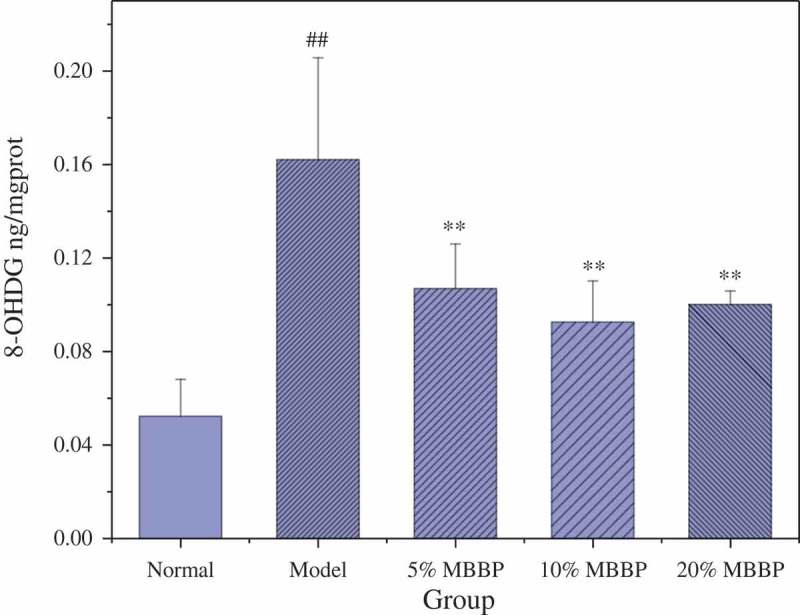
The effect of MBBP on the 8-OHDG level in diabetic mice. ## *p < *0.01 versus the normal group; ** *p < *0.01 versus the model group.

Reactive oxygen free radicals caused by oxidative stress in the body can lead to the production of a variety of chronic diseases. 8-OHdG, as the modified product of DNA oxidative damage, can be used to measure the degree of DNA oxidative damage and its repair level in the bio-organic body. To assess the degree of DNA damage, the 8-OHdG level was measured in liver tissue. In Figure 5, the 8-OHdG level in the model group was significantly higher than that of the normal group (*P* < 0.01), and showed that DNA oxidative damage became more prevalent in diabetic mice. In the MBBP treatment groups, the 8-OHdG level was significantly lower than that of the model group (*P* < 0.01) and decreased to 0.1 ng/mg. This suggested that MBBP can dramatically reduce the 8-OHdG level and significantly repair the DNA oxidative damage of diabetic mice.

### Organ coefficients

The mass ratio (tissue wet weight (mg)/BW (g)) of the mouse and its organs including the pancreas, liver, kidney, spleen, and testis after fasting are used to represent an organ coefficient that reflects swelling degree of organs. As shown in Table 1, the pancreas and liver coefficients of the model group were significantly higher than those of the normal group (*P* < 0.01). The main reason for this was that the body weights of diabetic mice were reduced significantly, and the results were consistent across the change in body weights (Figure 2). When the mice were dissected, we found that there were no obvious differences in the weight of the liver and pancreas between normal and model groups (data not listed). After treatment with MBBP, the pancreas and liver coefficients were significantly lower than those of the model group (*P* < 0.01 or *P* < 0.05); the pancreas and liver coefficients of the 20% MBBP group decreased significantly in comparison to the model group (*P* < 0.01). The results showed that diabetic symptoms were improved and were effectively relieved after treatment with MBBP in a dose-dependent manner.

**Table 1.** The effect of MBBP on the coefficients of organs to body weight.

Indexes (mg/g BW)						
Groups	BW (g)	Pancreas	Liver	Kidney	Spleen	Testis
Normal	48.35 ± 4.12	6.01 ± 0.77	43.1 ± 3.29	14.58 ± 2.42	2.72 ± 0.33	7.41 ± 0.1.49
Model	37.91 ± 3.87	7.41 ± 1.47^##^	60.93 ± 5.77^##^	22.44 ± 2.99^##^	3.51 ± 0.94^##^	7.66 ± 0.1.2
5% MBBP	40.38 ± 4.48	7.32 ± 1.63	59.65 ± 3.09	21.79 ± 1.84	3.25 ± 0.59	7.85 ± 0.1.66
10% MBBP	39.60 ± 3.45	7.14 ± 1.01	55.4 ± 3.78**	20.97 ± 2.72	3.15 ± 1.02	8.19 ± 0.1.49
20% MBBP	39.90 ± 2.89	6.18 ± 0.921*	53.87 ± 3.63**	17.94 ± 2.96**	2.65 ± 0.55**	8.15 ± 0.0.95

The kidneys and spleens of the diabetic mice appeared severely symptomatic; the kidney and spleen coefficients were significantly higher than for the normal group (*P* < 0.01); however, treatment with the MBBP reduced the coefficient effectively. The kidney and spleen coefficients of the 20% MBBP group decreased significantly compared to those of the model group (*P* < 0.01). The results showed that with an increasing MBBP dose, the treatment of the diabetic symptoms was effectively reduced in a dose-dependent manner. In addition, the testis coefficient of diabetic mice showed little difference compared with the normal group. With an increasing MBBP dose, the testis coefficient increased compared to that of the model group, but statistical analysis indicated that there were no significant differences between model and MBBP groups.

### Serum lipid level

To observe the effect of MBBP on these parameters, triglycerides (TG), HDL-C, cholesterol (CHOL), and LDL-C were tested. As shown in Table 2, TG levels in the model group were higher than those of the normal group. After four weeks of treatment with different doses of MBBP, the TG levels of the diabetic mice had improved significantly. TG levels of the 20% MBBP group were significantly lower than for the model group (*P* < 0.01). HDL-C is a sub-type of cholesterol that resists atherosclerosis and reduces the risk of coronary heart disease. As shown in Table 3, the serum HDL-C levels in the model group were significantly lower than the normal group (*P* < 0.01). After four weeks of MBBP treatment, there was significant amelioration of the serum HDL-C concentration, further supported by a dose-dependent increase in serum HDL-C level, peaking in the 20% MBBP group (*P* < 0.05). The CHOL level of the diabetic mice (model) was slightly higher than that of the normal group mice, but there were no significant statistical differences. In all three dose groups of the MBBP treatment, the CHOL level decreased and approached the levels seen in the normal group, with a value of 2.78 mmol/L. This test suggests that MBBP could reduce CHOL levels effectively. The decrease in serum LDL-C levels inversely correlated with the increase in serum HDL-C levels. Within the three different dose groups of MBBP, the LDL-C of the 5% MBBP group was dramatically lower than that of the model group (*P* < 0.01); its value approached 0.24 mmol/L. These results showed that the MBBP might regulate lipid metabolism effectively.

**Table 2.** The effect of MBBP on serum lipid levels of T2D mice.

	Serum lipid level (mmol/L)			
Groups	TG	HDL-C	CHOL	LDL-C
Normal	1.78 ± 0.447	2.81 ± 0.268	2.82 ± 0.416	0.242 ± 0.054
Model	2.04 ± 0.254	2.13 ± 0.075^##^	3.12 ± 0.131	0.39 ± 0.02^##^
5% MBBP	1.35 ± 0.437	2.31 ± 0.442	2.82 ± 0.571	0.265 ± 0.038**
10% MBBP	1.33 ± 0.98	2.38 ± 0.314	2.85 ± 0.544	0.357 ± 0.059
20% MBBP	1.35 ± 0.067**	2.46 ± 0.254*	2.78 ± 0.416	0.307 ± 0.032*

**Table 3.** Effect of MBBP on hepatic function of T2D mice.

Groups	AST (U/gprot)	ALT (U/gprot)
Normal	13.20 ± 3.03	12.52 ± 6.56
Model	34.82 ± 8.06^##^	20.92 ± 12.90
5% MBBP	25.55 ± 19.38*	18.31 ± 7.78
10% MBBP	23.37 ± 9.25**	14.24 ± 5.05
20%MBBP	15.19 ± 3.60**	13.61 ± 4.37

### Effect of the MBBP on the liver function

In the liver function tests, alanine aminotransferase (ALT) is used as the index for liver cell damage, and aspartate aminotransferase (AST) is the criterion for demonstrating liver cell necrosis. To study the effect of MBBP on liver function, both ALT and AST levels were measured. As shown in Table 3, AST levels in diabetic mice were significantly higher than for the normal group (*P* < 0.01). After the addition of MBBP, their AST levels were significantly reduced from 25.55 U/gprot to 15.19 U/gprot in comparison with the model group (34.82 U/gprot) (*P* < 0.05 or *P* < 0.01). The AST of the 20% MBBP group approached the normal group. ALT levels in the model mice were higher than those of the normal mice, but no statistically significant differences (*P* > 0.05) were found between any of the groups. After the addition of MBBP for four weeks, the ALT levels were gradually reduced in a dose-dependent manner. The results showed that the treatment of the 20% MBBP was the most effective, and in general, MBBP treatment improved liver cells and protected the liver.

### Antioxidant capacity

Under hyperglycemic conditions, the ability of the body to scavenge reactive oxygen free radicals (ROS) decreases, which leads to an accumulation of ROS and decreased antioxidant capacity. To evaluate the influence of MBBP on antioxidant capacity, the levels of T-SOD, GSH-PX, MDA, and T-AOC were investigated. The data in Table 4 show that the T-SOD level, GSH-PX level, and the T-AOC level of the mice in the model group decreased significantly compared to the normal group. After four weeks of treatment with three different MBBP doses, the T-SOD level, the GSH-PX level, and the T-AOC level were increased significantly, with a dose-dependent effect in comparison with the model mice. The level of T-SOD in the highest dose group reached 107.62 mmol/L, and the GSH-PX of the 20% MBBP group was significantly higher than that of the model group. The T-AOC of the 20% MBBP group approached that of the normal group and reached 0.831 mmol/L. The MDA level of diabetic mice was significantly higher than that of the normal mice. After four weeks of treatment with three different MBBP doses, MDA was decreased; the MDA of the 10% and 20% MBBP groups was significantly lower than that of the model group. The level (0.473 mmol/L) of MDA in the 20% MBBP group approached that seen in the normal group. These results suggest that MBBP may enhance antioxidant capacity effectively and reduce oxidative damage.

**Table 4.** The effect of MBBP on antioxidant capacity.

Groups	T-SOD (mmol/L)	GSH-PX (mmol/L)	MDA (mmol/L)	T-AOC (mmol/L)
Normal	113.19 ± 6.50	1469.6 ± 192.0	0.425 ± 0.169	0.836 ± 0.058
Model	98.05 ± 3.42^##^	1144.0 ± 26.2^##^	0.745 ± 0.120^##^	0.642 ± 0.032^##^
5% MBBP	104.99 ± 7.21**	1226.8 ± 241.5	0.635 ± 0.211	0.768 ± 0.082**
10% MBBP	103.71 ± 3.09**	1305.6 ± 255.4	0.507 ± 0.059**	0.807 ± 0.150**
20% MBBP	107.62 ± 4.77**	1437.2 ± 201.6**	0.473 ± 0.148**	0.831 ± 0.167**

## *p < *0.01 versus normal group; ** *p < *0.01 versus model group.

### Glucose and insulin tolerance

**Figure 6.**
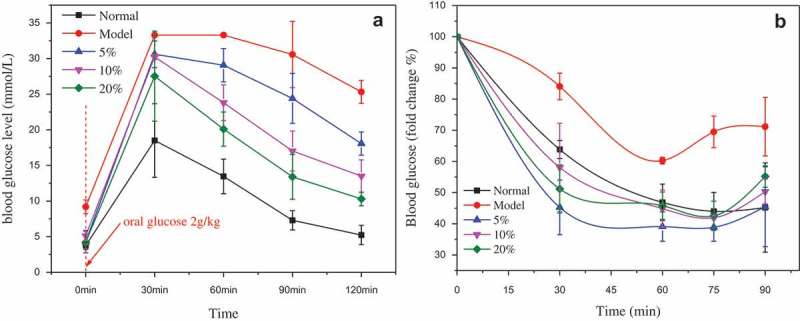
The effect of different doses of MBBP on OGTT (A) and ITT(B) in mice.

In the OGTT, after oral glucose, the blood glucose level was significantly lower at 30 min, 60 min, 90 min, and 120 min (Figure 6(a)). Interestingly, the decrease of blood glucose is consistent with the dose of MBBP; the blood glucose has evidently decreased with the high dose of MBBP in comparison with the lower dose of MBBP and is dose-dependent (Figure 6(a)), suggesting that the glucose-lowering effect of MBBP treatment is rapid and stable. Insulin resistance is demonstrated in the insulin tolerance test (ITT). In the ITT, the MBBP treatment reduced the blood glucose level at 30 min, 60 min, and 75 min (Figure 6(b)). High-dose MBBP improved insulin sensitivity, as demonstrated by lower blood glucose at 30–75 min following insulin injection, compared to the control mice.

### Pancreas pathologic tissue

**Figure 7.**
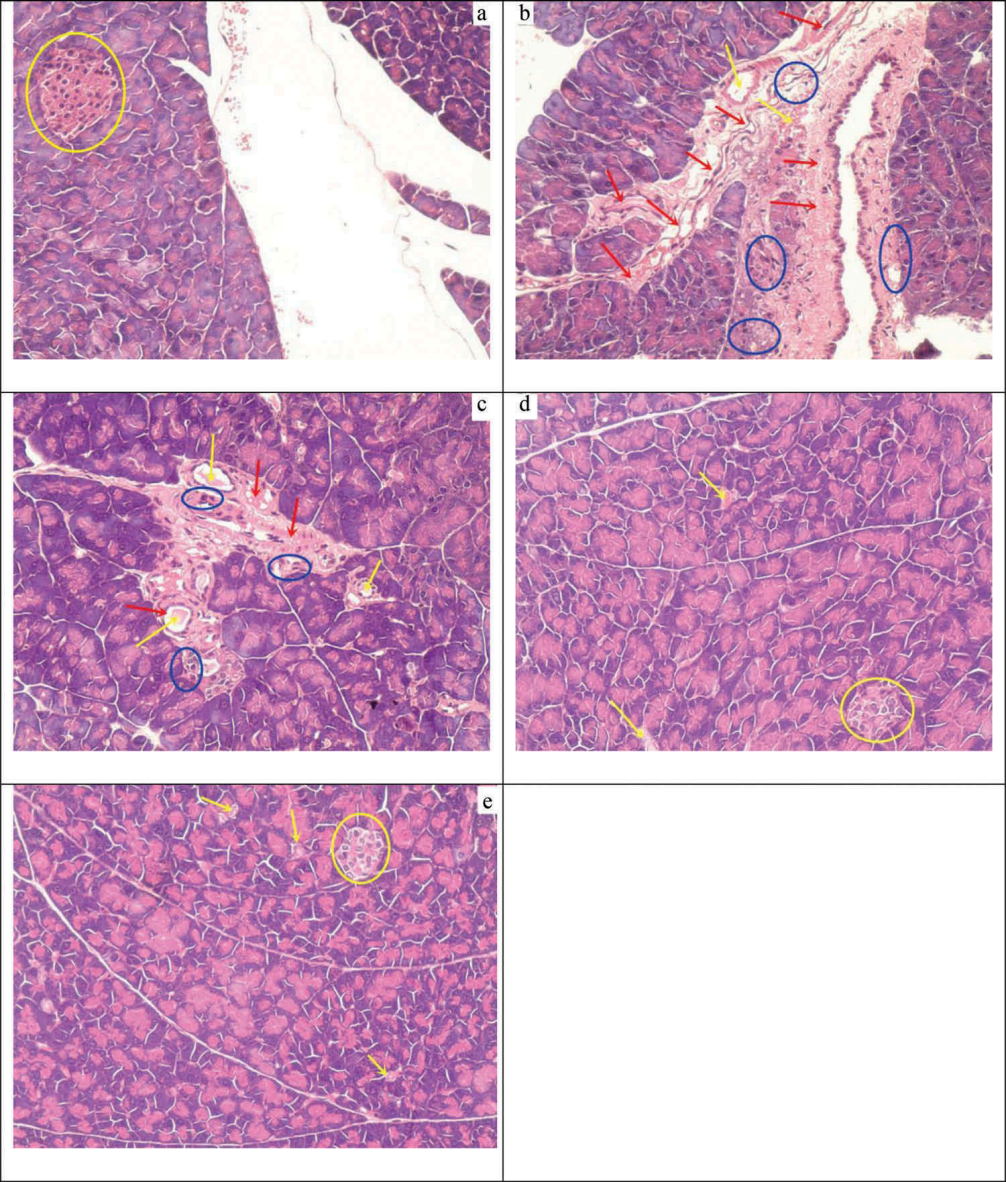
The effect of MBBP on the pathologic tissue in the pancreas.

Pancreatic cells were arranged compactly, and the cell nuclei were clearly visible in normal mice (Figure 7(a)). However, the pancreatic cells lost their normal structure and showed swelling, granular degeneration, local necrosis, focal necrosis, and karyolysis in diabetic mice (Figure 7(b)). When treated with different doses of MBBP, the pancreases of mice from the 5% MBBP group (Figure 7(c)) appeared to have moderate inflammatory cell infiltration, interstitial hyperplasia, and dilation of the blood vessels. The pancreases of mice from the 10% MBBP group (Figure 7(d)) appeared to have slight dilation of the blood vessels and congestion and hollowed out pancreas cells, but the cells were arranged compactly. It was possible to observe small, square, and complete islet structure. The pancreases of mice from the 20% MBBP group had no obvious pathological changes or hollowing. They showed slight dilation of blood vessels, the cells were arranged compactly, and the complete islet structure could be observed. The results showed that the pancreas of the diabetic mice treated with MBBP, especially in the 20% MBBP group, had a clear improvement, and were similar to those of the normal mice (Figure 7(e)).

### Insulin expression

**Figure 8.**
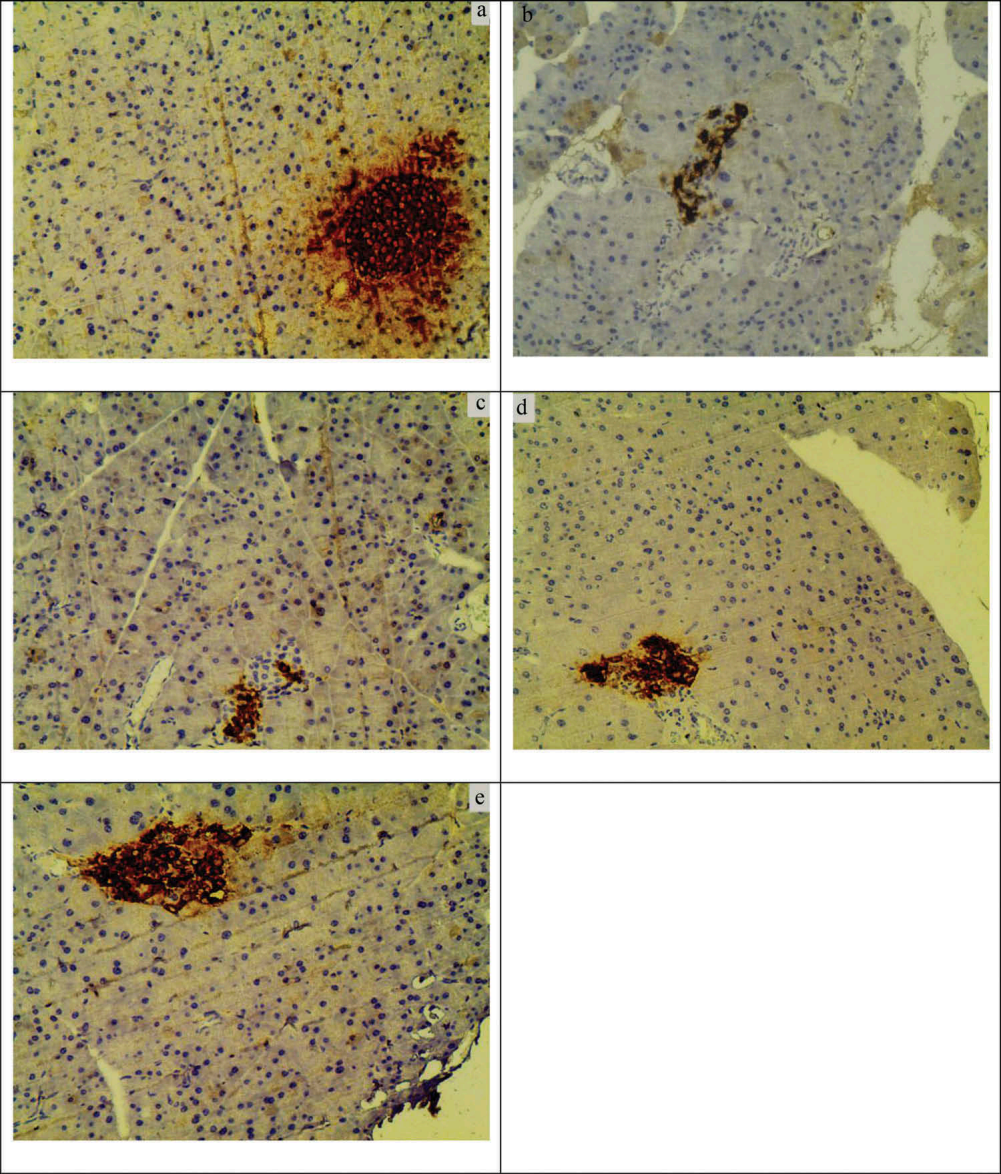
Insulin immunohistochemistry of pancreas β-cell in diabetic and normal mice. A: normal mice fed with a standard diet; B: model mice fed with a standard diet; C, D and E: treated mice fed with 5%, 10%, and 20% MBBP diets. Original magnification, ×400.

STZ can cause irreversible damage to pancreatic islet β-cells, inducing insulin secretion disorders, and resulting in the occurrence of diabetes [16]. The pictures in Figure 8 show that positive immunohistochemical staining was characterized by a deep tan in the pancreas; the positive parts mainly lying in the insulin-producing islet β-cells and the nuclei dyed blue. Figure 8(a) shows a deep tan region of the complete ellipse, with a smooth margin, coinciding with the islet structure. The secretion and site area was larger, and had tan particle distribution surrounded by a radial pattern, which suggested a normal islet structure and function and many rich β-cells with normal insulin secretion. Brown granules in the model group were significantly reduced and were almost not visible to the eye (Figure 8(b)), suggesting that β-cells were seriously damaged and had lost their normal structure and function. These granules were observed to gradually increase with MBBP treatment in a dose-dependent manner (Figure 8(c,d,e)), in comparison with the model group. The distribution area of the brown granules was larger and formed on oval shape in the 20% MBBP group. These results showed that oral administration of MBBP enabled the effective repair of the damaged β-cells in diabetic mice and improve the function of pancreatic islets.

### Expression of key enzymes in the liver

**Figure 9.**
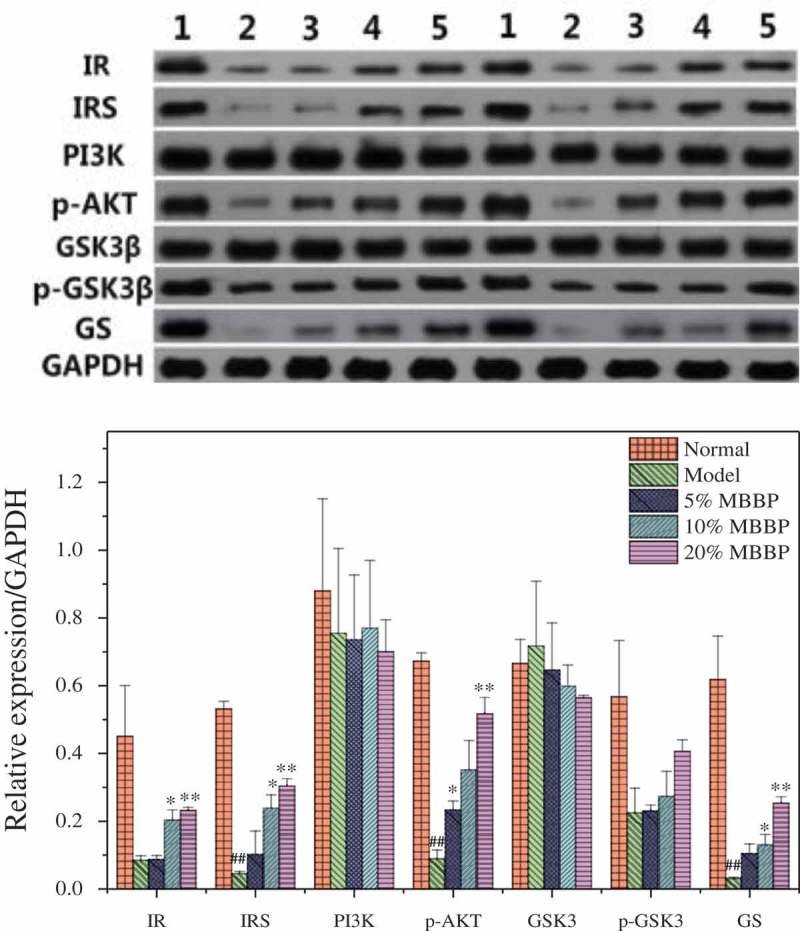
Protein levels of IR, IRS, PI3K, p-AKT, GSK3β, p-GSK3β, and GS in the liver by western blot. Density values were normalized to glyceraldehyde 3-phosphate dehydrogenase (GAPDH) levels. # *p < *0.05 and ## *p < *0.01 versus the normal group; * *p < *0.05 and ** *p < *0.01 versus the model group.

The IR, IRS, PI3K, P-AKT, GS, p-GSK3β, and GSK3β are important proteins in PI3K/AKT signaling a pathway about insulin secretion. As shown in Figure 9, the IR, IRS, P-AKT, and GS of the model group were significantly lower than for the normal group, which suggests that the transduction of insulin signaling and the synthesis of glycogen were inhibited in diabetic mice. The p-GSK3β of the MBBP-treated diabetic mice was higher than that of the model mice; it caused GS inactivation, which inhibited the synthesis of glycogen and glucose utilization in diabetic mice. After treatment with 5%, 10%, and 20% MBBP, the IR, IRS, P-AKT, and GS levels in the livers of mice from the MBBP groups were significantly increased compared with those in the normal group, and the highest dose group approached levels comparable to the normal group with a dose-dependent effect. The expression of PI3K could have increased through other factors, but the PI3K in the highest MBBP dose group was decreased. The reason may have been an experimental error. The expression of GSK3β was gradually decreased, and the expression of p-GSK3β was inversely increased. Therefore, the transduction of insulin enhanced the synthesis of glycogen and reduced the blood glucose level, eventually reversing diabetes.

### Glucose metabolism in the liver

**Figure 10.**
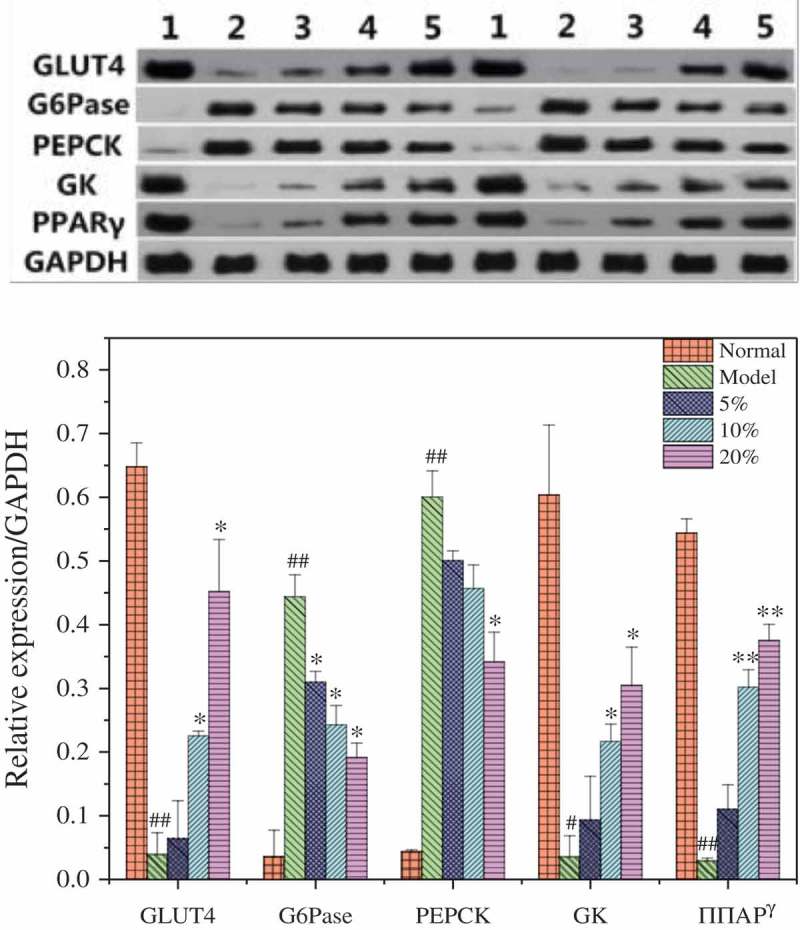
Protein levels of GLUT4, G6Pase, PEPCK GK, and PPARγ and in the liver by western blot. Density values were normalized to GAPDH levels. # *p < *0.05 and ## *p < *0.01 versus the normal group; * *p < *0.05 and ** *p < *0.01 versus the model group.

Under the stimulus of insulin, GLUT4 vesicles move to the cell membrane and fuse on the membrane; this affects glucose transportation [17]. Peroxisome proliferator-activated receptor (PPAR) is a key factor regulating adipocyte differentiation and fat metabolism. It has been shown that PPAR has an important regulatory function in glucose homeostasis [18]. PPARγ ligand activation can enhance the expression of GLUT4, and promotes the adipose tissue uptake of glucose, thereby improving sensitivity to insulin [19]. In Figure 10, the GLUT4 and PPARγ proteins of the model group are significantly lower than those of the normal group (*P* < 0.01), which suggests that sugar transportation was seriously inhibited in the livers of these mice. In the MBBP treatment groups, the protein levels of GLUT4 and PPARγ in the liver were increased significantly compared with those in the model group (*P* < 0.05 or *P* < 0.01). The level of PPARγ reached 0.4. Therefore, these data suggest that MBBP increased the expression of GLUT4 and PPARγ proteins and improved lipid metabolism and sugar transportation in the abnormal state of diabetic mice.

Both the use of sugar and its synthesis with G6Pase, PEPCK, and GK in the liver are inseparable. G6Pase and PEPCK are the key enzymes in the process of sugar dysplasia, high expression of which can cause high blood sugar. Increasing GK protein expression can lower blood sugar. The data in Figure 10 show that the G6Pase and PEPCK expression were increased and GK expression decreased in the model group in comparison with the normal group. With different doses of MBBP treatment, the G6Pase and PEPCK expression was decreased (*P* < 0.05) and the GK expression increased (*P* < 0.05). The protein expression had an obvious dose-dependent effect. The results showed that MBBP decreased the synthesis of blood glucose, enhanced the utilization of blood glucose, and consequently decreased the blood glucose level in the liver.

### Inflammatory factors in the liver

**Figure 11.**
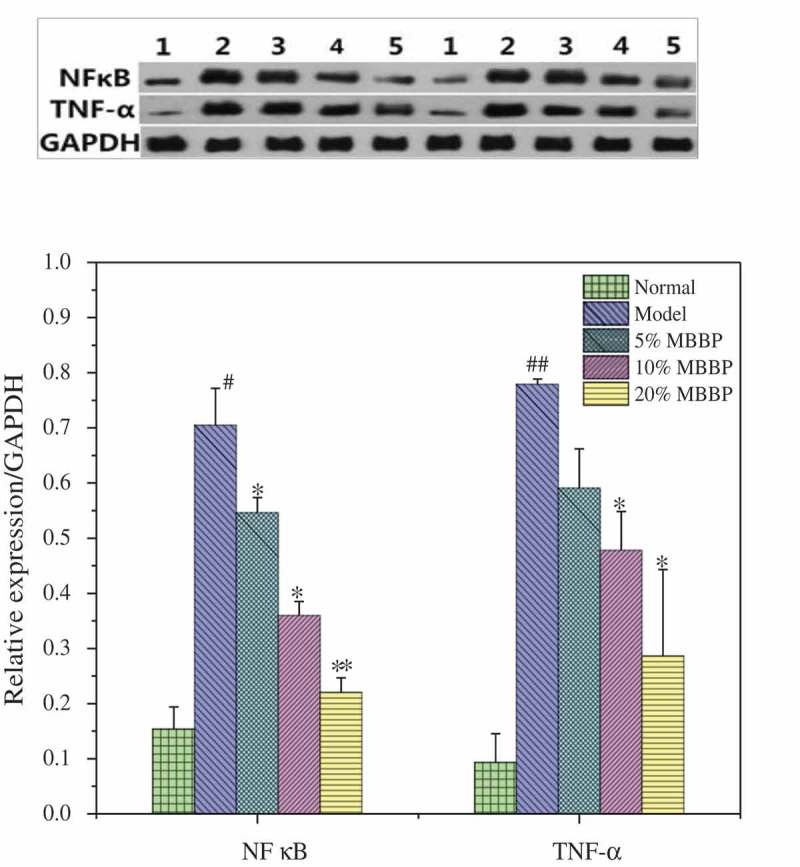
Protein levels of NF-κB and TNF-α in the liver by western blot. Density values were normalized to GAPDH levels. # *p < *0.05 and ## *p < *0.01 versus the normal group; * *p < *0.05 and ** *p < *0.01 versus the model group.

NF-κB is the key factor in a variety of inflammatory responses. The nonenzymatic saccharification of protein, the later saccharification product, and excessive reactive oxygen species can activate the NF-κB under diabetic conditions [20]. Activated NF-κB induces gene transcription and expression of TNF-α, interleukin, etc. [21]. TNF-α can inhibit the transportation of glucose and interfere with the transduction of insulin signaling [22]. Figure 11 shows that the TNF-α expression in the model group was significantly increased compared with those of the normal mice (*P* < 0.05 or *P* < 0.01), which suggested that the liver had severe inflammation. When treated with different doses of MBBP, the NF-κB and TNF-α levels were gradually decreased and approached that of the normal group; the TNF-α level was significantly decreased from 0.75 to 0.25 (*P* < 0.05). This result showed that MBBP could decrease the NF-κB and TNF-α level in diabetic mice, thus relieving inflammation and improving liver function.

### Insulin secretion in the pancreas

**Figure 12.**
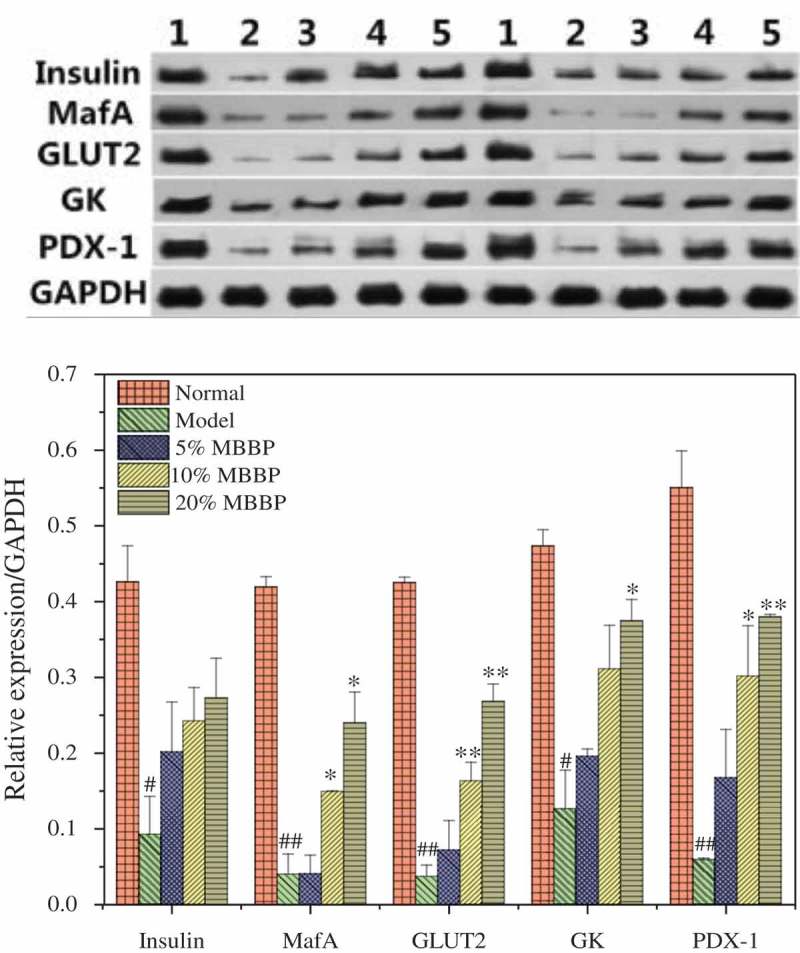
Protein levels of insulin, MafA, GLUT2, GK, and PDX-1 in the pancreas by western blot. Density values were normalized to GAPDH levels. # *p < *0.05 and ## *p < *0.01 versus the normal group; * *p < *0.05 and ** *p < *0.01 versus the model group.

MafA is a specific β-cell nuclear factor that regulates the expression of insulin as a transcription factor [23]. MafA can not only regulate insulin expression, but can also play a regulatory role for other related factors such as GLUT2, glucokinase (GK), and PDX-1. GLUT2 exists in islet β-cells; it is a kind of transmembrane transporter, which can transport glucose, adjust the membranes of ion channels, and promote the secretion of insulin. Phosphorylation of GK could catalyze glucose in islet β-cells. Long-term high blood sugar could lead to a decline in the activity of GK, thus affecting normal glucose metabolism in β-cells. PDX-1 plays a key role in pancreatic cells in the early stage of differentiation and in the process of maturation and regeneration. As shown in Figure 12, the insulin MafA, GLUT2, GK, and PDX-1 levels of the model group were lower than those of the normal group (*P* < 0.05 or *P* < 0.01). This showed that regulatory factors related to insulin became imbalanced in diabetic mice, leading to insufficient insulin secretion and turbulence in sugar transport and metabolism. With different doses of MBBP treatment, the insulin MafA, GLUT2, GK, and PDX-1 levels were increased with a dose-dependent effect in comparison with the model group. The MafA, GLUT2, GK, and PDX-1 levels in the 20% MBBP group were significantly higher than those of the other groups. MBBP could significantly improve the regulation of the expression of these factors, and promote insulin secretion and sugar metabolism.

### Islet cell apoptosis

**Figure 13.**
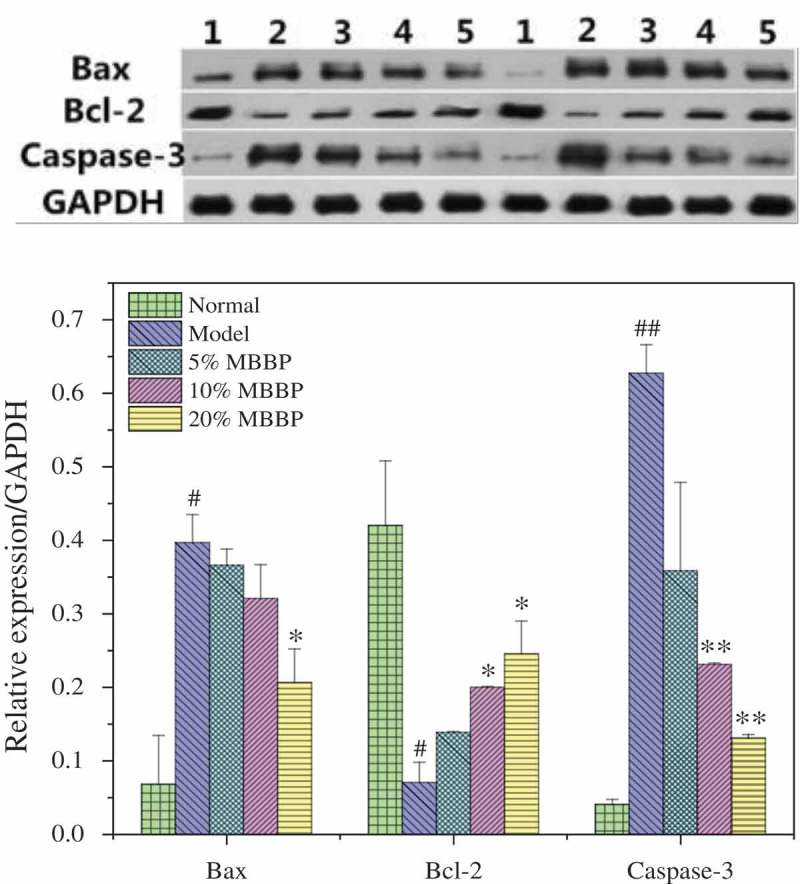
Protein levels of Bax, Bcl-2, and Caspase-3 in the liver. Density values were normalized to GAPDH levels. # *p <*0.05 and ## *p <*0.01 versus the normal group; * *p <*0.05 and ** *p <*0.01 versus the model group.

Bax is a cell factor that promotes apoptosis; activated Bax is induced in the cytoplasm and then activates the apoptotic gene Caspase-3. Bcl-2 is a mitochondrial protein, and can resist the promotion of apoptosis by Bax. High expression of Bcl-2 can also effectively inhibit the activation and apoptosis of Caspase-3 factors. In Figure 13, WB results show that the Bax and Caspase-3 levels in the pancreases of mice from the model group were significantly higher than those of the normal group (*P* < 0.05 or *P* < 0.01), and the Bcl-2 level was lower than that of the normal group. It showed that the model group incurred serious apoptosis; this result is consistent with the observation in above pathological pancreas. With the different doses of MBBP treatment, the Bax and Caspase-3 levels decreased and the Bcl-2 level increased significantly in the pancreas of mice in the model group. The levels of Bax, Caspase-3, and Bcl-2 differed in comparison with the model group, especially in the highest dose group. For example, the level of Caspase-3 in the model group was much higher (0.62) than that of the 20% MBBP group. This suggests that MBBP can downregulate the expression of these apoptotic factors, inhibit apoptosis of pancreatic β-cells, and maintain the structure and function of the pancreas.

## Discussion

This experiment, based on a previous study on *R. mori*, introduces whole mulberry powder into the diet to treat diabetic mice. It keeps the active ingredients of *R. mori* to the maximum extent and avoids complicated extraction processing, which causes the functional decrease of the bioactive components. There have been reports that diets rich in fiber could effectively reduce the FBG value of diabetic patients [24,25]. In this experiment, MBBP has more than 50% of the fiber of mulberry branch bark. It is very possible that the dietary fiber also has an auxiliary effect on lowering the FBG of the diabetic mice.

The FBG of the diabetic mice was always maintained at a higher level. After MBBP addition, FBG levels gradually declined with an evident dose-dependent effect. The biggest decrease of FBG was obvious in the 20% MBBP group. Because of damage of pancreas cells from STZ, the model group appeared more severely insulin resistant. After oral administration of MBBP, however, insulin levels significantly declined; the 20% MBBP treatment had the best effect on the improving insulin resistance. STZ increased ROS and declined oxidation resistance in mice. In diabetes, excess ROS in the body attacks DNA and produces 8-OHdG as an indicator of DNA oxidative damage [26]. Our results showed 8-OHdG and MDA levels in T2D mice far beyond those in the normal group, which is consistent with the results by Pan et al. [27]. Our previous researches reported that MBBP and the extraction of MBBP can decrease blood glucose in vivo and in vitro by inhabiting α-Glycosidase to stop amylohydrolysis. Besides, others flavonoid compounds in MBBP can enhanced anti-oxidative capacity [8]. So the level enhancements of SOD, GSH-PX, and T-AOC suggest that MBBP can reduce the degree of DNA damage in diabetic mice by enhancing antioxidant capacity.

Sustained high level of blood sugar can lead to microvascular lesions and cause circulation disorders in the liver [28]. ALT and AST are important indicators of liver function. The two enzymes were significantly higher in the model group than in the normal group. After treatment with MBBP, these levels gradually reduced. The MBBP can effectively reduce liver tissue damage and protect the liver function. Further, lipid metabolism in diabetic mice was abnormal, but after treatment with MBBP, TG, CHOL, and LDL-C significantly decreased and HDL-C increased, which is consistent with the results described by Kim et al. [29]. MBBP can effectively improve dyslipidemia and regulate lipid metabolism in diabetic mice.

Oxidative stress can cause damage in a variety of organs, such as non-alcoholic fatty liver disease [30], kidney disease [31], and pathological testicular changes [32]. Oxidative stress is likely to be the main mechanism by which high blood glucose causes toxic injury to β-cells [33]. Islet β-cells are very prone to apoptosis after oxidative damage; previous studies have proved that oxidative stress can directly cause β-cell apoptosis in the mitochondria [34]. Our H&E staining and immunohistochemical results showed that the pancreas of model mice appeared to be severely damaged, and intact islet β-cells were most difficult to find. After therapy with MBBP, some islet β-cells were easily observed, and the normal function of these β-cells secreting insulin was also gradually restored.

OGTT is a kind of criterion to judge glucose tolerance. In this test, the blood glucose level in normal and MBBP-fed mice recovers the previous level in the normal range, but the blood glucose level is still high in diabetic mice, suggesting the treatment of MBBP would increase glucose tolerance. Insulin resistance is an early condition of metabolic disorders, which could lead to diabetes, hyperlipidemia, and steatosis [35]. For ITT, MBBP administration improved insulin sensitivity in the MBBP group mice, compared to the diabetic mice.

In insulin signaling pathways, disorder of any linkage with a receptor could result in insulin secretion [36]. The protein factors involved in insulin transduction are abnormal, thus it is likely to lead to signal blockage or reduced signaling and cause disorder of the entire pathway [37]. When the insulin combines with the IR outside the cell membrane and consequently activates PI3K via IRS, the activated PI3K further activates p-AKT via a series of signaling molecules. p-AKT can then phosphorylate GSK3β, which deactivates it. Although GSK3β is commonly described as a key enzyme in a plethora of signaling cascades, it was originally identified as playing an important role in the regulation of glycogen synthesis because of its ability to inactivate GS by phosphorylation. Acting as a constitutively active kinase, GSK3β phosphorylates GS, which results in a decrease in glycogen production [38], and thus, it reduces the synthesis of glycogen. The phosphorylation of GSK3β can promote the synthesis and storage of glycogen; the dephosphorylation of GSK3β can reduce the synthesis of glycogen. The experimental results from this study have shown that the expression levels of IR, IRS, p-AKT, p-GSK3β, GS, GK, GLUT4, and PPARγ were significantly decreased, while those of GSK3β, G6Pase, and PEPCK were increased significantly in the liver, indicating insulin signal transduction, glucolipid metabolism, and glycogen synthesis appears to be blocked in diabetic mice, leading to a decrease in glucose utilization rate and a subsequent increase in blood sugar. After MBBP treatment, the expression levels of these factors were obviously improved, especially in the 20% MBBP group in which both levels of GLUT4 and PPARγ were increased significantly in the liver. PPARγ has high expression in adipocytes and plays an important role in liver lipid storage and differentiation of fat cells. Lee et al. have earlier suggested that PPARγ ligand upregulated the expression of genes involved in glucose uptake and lipid storage of adipocytes as well as lipid uptake and storage of the liver [39]. GLUT4 plays an explicit role in regulation of glucose homeostasis through translocation and activation, subsequently triggered by the insulin dependent phosphatidylinositol 3-kinase (PI3K)/phosphorylated protein kinase B (p-Akt) pathway [40,41]. PPARγ ligands after activation can promote adipose tissue by enhanced expression of GLUT4 for glucose uptake [19,42]. The activated PPARγ is possible to enhance the conduction of insulin signal and regulate the endocrine of adipose cells [43]. It is well known that the mulberry or its branch bark has lots of active components such as alkaloids, flavonoids, phenols, and saccharides. Recently, our group has revealed in both in vitro and in vivo assays that the branch bark ethanol extract strongly inhibited both α-glucosidase and sucrase activities. It could effectively inhibit the postprandial hyperglycemia as a novel α-glucosidase activity inhibitor for diabetic therapy [8,11]. Decades ago, it had been found that 1-deoxynojirimycin (DNJ) was one of the alkaloids in mulberry strongly inhibiting α-glucosidase both in vitro and in vivo [44,45]. It was also reported that the hybrid of DNJ and polysaccharide from mulberry leaves may have antidiabetic activity by regulating the expression of the hepatic gluconeogenesis enzymes, glucokinase, phosphoenolpyruvate carboxykinase, and glucose-6-phosphatase [46]. Its analog acarbose has been commercialized and used clinically for many years. Besides, mulberry and mulberry branch bark include many flavonoids and phenols, such as quercetin, kaempferol, glucoside, and mulberroside A, and they all have an antioxidant effect and inhibit α-glucosidase and sucrase activities [12]. Therefore, the upregulation of GLUT4 and PPARγ may be in relation to many active components of MBBP. The detailed molecular mechanisms of GLUT4 and PPAR-gamma will be researched in the upcoming work.

Our inflammation tests also showed that NF-κB and TNF-α appeared abnormally increased, which suggests that the liver suffers serious inflammation. With MBBP treatment, the levels of both factors decreased significantly; the liver inflammation was also obviously decreased. In addition, the experimental results also illustrated that the MBBP can increase the MafA, GLUT2, GK, and PDX-1 protein expressions in the pancreas of diabetic mice, which promote insulin secretion and thus strengthen glucose metabolism. MBBP also reduces the expression of the pro-apoptosis factors and Caspase-3 in the pancreas and increases the expression of the anti-apoptotic factor Bcl-2. It retards apoptotic damage to pancreatic cells and protects the structure and function of the pancreas cells.

In general, oral administration of MBBP regulated insulin secretion and effectively maintained normal levels of glucose metabolism in mice, which may be realized through improving the antioxidant capacity, inhibiting apoptosis of pancreas cells, and repairing liver and pancreas injury.

## References

[1] Grant RW, Moore AF Florez JC. Genetic architecture of type 2 diabetes: recent progress and clinical implications. Diabetes Care. 2009;32 (6):1107–16. doi:10.2337/dc08-2171 19460916 PMC2681026

[2] Guo MH. Literature analysis of the low blood sugar reactions caused by glibenclamide. Chin J Pharmacovigilance. 2013;10:424–431.

[3] Singaba ANB, El-Beshbishyb HA, Makiko Y, et al Hypoglycemic effect of Egyptian *Morus alba* root bark extract: effect on diabetes and lipid peroxidation of streptozotocin-induced diabetic rats. J Ethnopharmacol. 2005;100(3):333–338. 15885940 10.1016/j.jep.2005.03.013

[4] Sendrayaperumal V, Iyyam Pillai S, Subramanian S Design, synthesis and characterization of zinc–morin, a metal flavonol complex and evaluation of its antidiabetic potential in HFD-STZ induced type 2 diabetes in rats. Chem Biol Interact. 2014;219:9–17. 24854284 10.1016/j.cbi.2014.05.003

[5] Andallu B, Radhika B, Suryakantham V Effect of *aswagandha*, ginger and mulberry on hyperglycemia and hyperlipidemia. Plant Foods Hum Nutr. 2003;58:1–7.12859008

[6] Hamdy SM Effect of *Morus Alba* Linn extract on enzymatic activities in diabetic rats. J Appl Sci Res. 2012;8(1):10–16.

[7] Eun Jung K, Yeon LJ, Sook C Physicochemical properties and antioxidant activities of korean traditional alcoholic beverage, Yakju, enriched with mulberry. J Food. 2012;77(7):752–758. 10.1111/j.1750-3841.2012.02753.x 22671858

[8] Liu HY, Fang M, Zhang YQ In vivo hypoglycaemic effect and inhibitory mechanism of the branch bark extract of the mulberry on STZ-induced diabetic mice. Scientific World Journal. 2014; 2014:614265. 10.1155/2014/614265 PMC4142180 25177729

[9] He TZ, Li L, Liu XM, et al Extraction, composition and in vitro bioactivity of polysaccharide from mulberry branch bark. Science Sericulture (In Chinese). 2010;36(6):1033–1036.

[10] H Y L, Wang J, Ma J, et al Interference effect of oral administration of mulberry branch bark powder on the incidence of type II diabetes in mice induced by streptozotocin. Food Nutr. 2016;60:31606. 10.3402/fnr.v60.31606 PMC4891971 27257845

[11] Wang S, Fang M, Yong-Lei M, et al Preparation of the branch bark ethanol extract in mulberry Morus alba, its antioxidation, and antihyperglycemic activity in vivo. Evidence-Based Complement Altern. 2014;2014:569652. 10.1155/2014/569652 PMC3920605 24587809

[12] Wang S, Liu X-M, Zhang J, et al An efficient preparation of mulberroside A from the branch bark of mulberry and its effect on the inhibition of tyrosinase activity. PLOS One. 2014;9:e109396. 25299075 10.1371/journal.pone.0109396 PMC4192315

[13] Wan L-Z, Bin M, Zhang Y-Q Preparation of morusin from Ramulus mori and its effects on mice with transplanted H22 hepatocarcinoma. Biofactors. 2014;40(6):636–645. 25422054 10.1002/biof.1191

[14] Ding B, Lv Y, Zhang Y-Q Anti-tumor effect of morusin from the branch bark of cultivated mulberry in Bel-7402 cells via the MAPK pathway. RSC Adv. 2016;6:17396–17404.

[15] Qiu F, Tian-Zhen H, Zhang Y-Q Two polysaccharides isolated from mulberry branch bark and their antioxidant activity. Archi Pharm Res. 2016;39(5):887–896. 10.1007/s12272-016-0742-8 27255450

[16] Norquay LD, D’Aquino KE, Opare-Addo LM, et al Insulin receptor substrate-2 in β-cells decreases diabetes in nonobese diabetic mice. Endocrinology. 2009;150:4531–4540. 19574401 10.1210/en.2009-0395 PMC2754683

[17] Rea S, James DE Moving GLUT4: the biogenesis and trafficking of GLUT4 storage vesicles. Diabetes. 1997;46:1667–1677. 9356011 10.2337/diab.46.11.1667

[18] Siddiqui AM, Cui X, Wu R, et al The anti-inflammatory effect of curcumin in an experimental model of sepsis is mediated by up-regulation of peroxisome proliferator-activated receptor-γ. Cri Care Med. 2006;34:1874–1882 10.1097/01.CCM.0000221921.71300.BF 16715036

[19] Hamm JK, Jack AKE, Pilch PF, et al Role of PPARγ in regulating adipocyte differentiation and insulin-responsive glucose uptake. Ann New York Acad. 1999;892:134–145. 10.1111/j.1749-6632.1999.tb07792.x 10842659

[20] Yang XH, Cui W, Wang YY Atorvastatin on type 2 diabetic rats myocardial NF-kappa B, the influence of the expression of TNF-α. Journal Med People’s Liberation Army. 2008;33(3):282–285.

[21] Aragno M, Mastrocola R, Medana C, et al Oxidative stress-dependent impairment of cardiac-specific transcription factors in experimental diabetes. Endocrinology. 2006;147(12):5967–5974. 16935841 10.1210/en.2006-0728

[22] Wang ZJ, Zhang Y, Su J TNF-α in diet induced diabetic rats, the expression of the liver and the intervention of rosiglitazone study. J Hebei. 2007;29(1):33.

[23] Moates JM, Nanda S, Cissell MA, et al BETA2 activates transcription from the upstream glucokinase gene promoter in islet beta-cells and gut endocrine cells. Diabetes. 2003;52(2):403–408. 12540614 10.2337/diabetes.52.2.403

[24] Kay RM, Grobin W, Track NS Diets rich in natural fibre improve carbohydrate tolerance in maturity-onset, non-insulin dependent diabetics. Diabetologia. 1981;20(1):18–21. 6259009 10.1007/BF00253811

[25] Jenkins DJA, Taylor RH, Wolever TMS The diabetic diet, dietary carbohydrate and differences in digestibility. Diabetologia. 1982;23(6):477–484. 6295862 10.1007/BF00254294

[26] Maiko K, Toyoshi I, Toshiyo S, et al Accumulation of 8-hydroxy-2ʹ-deoxyguanosine and mitochondrial DNA deletion in kidney of diabetic rats. Diabetes. 2002;51:1588–1595. 11978660 10.2337/diabetes.51.5.1588

[27] Pan HZ, Chang D, Feng LG, et al Oxidative damage to DNA and its relationship with diabetic complications. Biomed Environ. 2007;20(2):160–163. 17624192

[28] Santos VP, Caffaro RA, Pozzan G, et al Comparative histological study of atherosclerotic lesions and microvascular changes in amputated lower limbs of diabetic and non-diabetic patients. Arq Bras Endocrinol Metabol. 2008;52(7):1115–1123. 19082299 10.1590/s0004-27302008000700007

[29] Kim K, Kim H, Kwon J, et al Hypoglycemic and hypolipidemic effects of processed Aloe vera gel in a mouse model of non-insulin-dependent diabetes mellitus. Phytomedicine. 2009;16:856–863. 19303272 10.1016/j.phymed.2009.02.014

[30] Brouns R, Deyn PPD The complexity of neurobiological processes in acute ischemic stroke. Clin Neurol Neurosurg. 2009;111(6):483–495. 19446389 10.1016/j.clineuro.2009.04.001

[31] Yoh K, Hirayama A, Ishizaki K, et al Hyperglycemia induces oxidative and nitrosative stress and increases renal functional impairment in Nrf2-deficient mice. Genes to Cells. 2008;13(11):1159–1170. 19090810 10.1111/j.1365-2443.2008.01234.x

[32] Altay B, Cetinkalp S, Doganavsargil B, et al Streptozotocin induced diabetic effects on spermatogenesis with proliferative cell nuclear antigen immunostaining of adult rat testis. Fertil Steril. 2003;80(2):828–831. 14505760 10.1016/s0015-0282(03)00984-1

[33] Robertson RP Chronic oxidative stress as a central mechanism for glucose toxicity in pancreatic islet beta cells in diabetes. J Biol Chem. 2004;279:42351–42354. 15258147 10.1074/jbc.R400019200

[34] Fariss MW, Chan CB, Patel M, et al Role of mitochondris in toxic oxidative stress. Mol Interv. 2005;5:94–111. 15821158 10.1124/mi.5.2.7

[35] Fan S, Zhang Y, Sun Q, et al Extract of okra lowers blood glucose and serum lipids in high-fat diet-induced obese C57BL/6 mice. J Nutr Biochemistry. 2014;25(7):702. 10.1016/j.jnutbio.2014.02.010 24746837

[36] Lobo S, Bernlohr DA Fatty acid transport in adipocytes and the development of insulin resistance. Novartis Found Symp. 2007;286(286):113–126. 18269178 10.1002/9780470985571.ch10

[37] Prudente S, Morini E, Trischitta V Insulin signaling regulating genes: effect on T2DM and cardiovascular risk. Nat Rev Endocrinol. 2009;5(12):682–693. 19924153 10.1038/nrendo.2009.215

[38] Marcos EF Putative role of glycogen as a peripheral biomarker of GSK3β activity. Med Hypotheses. 2013;81:376–378. 23809426 10.1016/j.mehy.2013.05.020

[39] Park MY, Lee KS, Sung MK Effects of dietary mulberry, Korean red ginseng, and banaba on glucose homeostasis in relation to PPAR-alpha, PPAR-gamma, and LPL mRNA expressions. Life Sci. 2005;77:3344–3354. 15979095 10.1016/j.lfs.2005.05.043

[40] Watson RT, Kanzaki M, Pessin JE Regulated membrane trafficking of the insulin- responsive glucose transporter 4 in adipocytes. Endocr Rev. 2004;25:177–204. 15082519 10.1210/er.2003-0011

[41] Gandhi GR, Stalin A, Balakrishna K, et al Insulin sensitization via partial agonism of PPARγ and glucose uptake through translocation and activation of GLUT4 in PI3K/p-Akt signaling pathway by embelin in type 2 diabetic rats. Biochim Biophys Acta. 2013;1830:2243–2255. 23104384 10.1016/j.bbagen.2012.10.016

[42] Naowaboot J, Pannangpetch P, Kukongviriyapan V, et al Mulberry leaf extract stimulates glucose uptake and GLUT4 translocation in rat adipocytes. Am J Chin Med. 2012;40:163–175. 22298456 10.1142/S0192415X12500139

[43] Kurosaki E, Nakano R, Shimaya A, et al Differential effects of YM440 a hypoglycemic agent on binding to a peroxisome proliferator-activated receptor gamma and its transactivation. Biochem Pharmacol. 2003;65:795. 12628477 10.1016/s0006-2952(02)01617-9

[44] Nojima H, Kimura I, Chen F-J, et al Antihyperglycemic effects of N-containing sugars from *Xanthocercis zambesiaca, Morus bombycis, Aglaonema treubii*, and *Castanospermum australe* in streptozotocin-diabetic mice. Nat Prod. 1998;61:397–400. 10.1021/np970277l 9544568

[45] Fang M, Huang AQ, Zhang YQ The correlation between 1-deoxynojirimycin content and α-glucosidase inhibitory activity in the bark ethanol extract from *Ramulus mori* . 2012; International Conference on Biomedical Engineering and Biotechnology; May 28–30, Macau, China, 1795–1798.

[46] Li YG, Ji D-F, Zhong S, et al Hybrid of 1-deoxynojirimycin and polysaccharide from mulberry leaves treat diabetes mellitus by activating PDX-1/insulin-1 signaling pathway and regulating the expression of glucokinase, phosphoenolpyruvate carboxykinase and glucose-6-phosphatase in alloxan-induced diabetic mice. J Ethnopharmacol. 2011;134:961–970. 21333726 10.1016/j.jep.2011.02.009

